# Clinicopathological characteristics of unicentric retroperitoneal Castleman’s disease: a study of 14 cases

**DOI:** 10.1186/s12957-015-0756-6

**Published:** 2016-01-06

**Authors:** Shuai Wang, Shanwen Chen, Jiangfeng Xu, Songliang Cai

**Affiliations:** 1 General Surgery, The fourth affiliated hospital Zhejiang University School of Medicine, Shangcheng Road NO.N1, Yiwu, Zhejiang 322000 China; 2 Urology Surgery, The first affiliated hospital Zhejiang University School of Medicine, Qingchun Road No. 79, Hangzhou, Zhejiang 310000 China

**Keywords:** Unicentric retroperitoneal Castleman’s disease, Surgery, Clinicopathological characteristics

## Abstract

**Background:**

The objectives of this study are to investigate the clinicopathological characteristics and prognosis analysis of unicentric retroperitoneal Castleman’s disease (CD), and to improve the level of diagnosis and treatment of unicentric retroperitoneal CD.

**Methods:**

The clinical data of 14 patients with unicentric retroperitoneal CD undergoing surgery from September 2007 to March 2014 were retrospectively reviewed.

**Results:**

There were six males and eight females with a median age of 39 years old (range 15–58). Only three patients had a clinical manifestation of abdominal pain, and one patient associated with myasthenia gravis. All patients underwent surgical resection. The mean operation time was 137 min with a range of 72–472 min. The mean blood loss was 143 ml (range 50–500 ml). The CD was confirmed by histopathology. There were hyaline vascular (HV) type of CD in 13 cases, and mixed type of CD in one case. The mean hospital stay was 17.9 days with a mean postoperation hospital stay of 9.2 days. The duration of follow-up ranged from 21 to 99 months for 14 cases. All the 14 patients were alive without recurrence.

**Conclusions:**

Unicentric retroperitoneal CD is a rare disease that is often misdiagnosed due to the absence of specific clinical manifestations. The final diagnosis depends on pathologic examination. Complete surgical resection of the tumor is the best therapeutic alternative for unicentric CD.

## Background

Castleman’s disease (CD) was first described in 1956 by Benjamin Castleman, who identified a series of patients with solitary hyperplastic mediastinal lymph nodes containing small, hyalinized follicles, and marked interfollicular vascular proliferation [1]. The etiology is unknown, and it is characterized by the development of tumorous masses of lymphoid tissue. Two clinical types (unicentric and multicentric) have been subclassified on histological forms: hyaline vascular (HV) type, plasma cell (PC) type, and mixed type. As this disease is rare and poorly understood, the optimal therapy is unknown. This study was conducted to analyze the clinicopathological characteristics and surgical treatment for patients with unicentric retroperitoneal CD.

## Methods

From September 2007 to October 2015, a total of 14 patients with unicentric retroperitoneal CD were managed at our hospital. These patients were retrieved from our pathology database, and the medical records were reviewed for demographic data, clinical and pathological characteristics. The extent of the disease was assessed with the help of ultrasonography (US) and computed tomography (CT) of the abdomen and routine blood chemistry analyses. The diagnosis of CD was made based on the postoperative histological examination. Pathologically, hyaline vascular CD had hyaline vascular follicles and interfollicular capillary proliferation. Plasma cell CD was characterized by follicular hyperplasia with intervening sheets of plasma cells. If the features of both types were present, it was diagnosed as mixed type. The diagnosis of these types was based on the criteria of Keller et al. [2].

The end-points were survival and recurrence. Follow-up information was obtained. All patients were either followed in our clinic or by telephone contact anywhere possible until the time of the patients’ death or till the end of this study. The duration of follow-up was defined as the interval between diagnosis and the last contact with each patient. Local recurrence was defined as tumor relapse within the region of operative files.

## Results

The study included all 14 cases of histologically confirmed CD at our hospital from September 2007 to March 2014. The relevant clinical findings of all the 14 cases are summarized in (Table 1). Eight patients (57 %) were female and six (43 %) were male, with a median age of 39 years old and a range of 15–58 years. Ten patients were asymptomatic, and masses were identified either incidentally or by physical examination. Only three patients had a clinical manifestation of abdominal pain or uncomfortable, and one patient associated with myasthenia gravis. The medical history and laboratory findings of these patients were unremarkable. The tumor marker of all cases, including CA-125, CA-19-9, CEA, AFP, were normal. The tumor size determined by either US or CT ranged from 3.0 × 2.5 × 1.8 to 7.0 × 7.5 × 5.0 cm. In all the patients, the diagnosis of CD was confirmed after the resection and histological examination of the specimen. Thirteen patients had HV type and one patient had mixed type.

**Table 1:** Patients and treatment, and survival in unicentric retroperitoneal CD

Patient (*n*)	Age (years)	Sex	Histological type	Treatment	Hospital stay (days)	Follow-up (months)	Outcome
1	39	F	HV	CR	15	80	A-NED
2	30	M	HV	CR	20	61	A-NED
3	31	F	HV	CR	18	59	A-NED
4	15	F	HV	CR	13	46	A-NED
5	44	M	HV	CR	26	40	A-NED
6	58	F	HV	CR	12	40	A-NED
7	32	F	HV	CR	21	33	A-NED
8	40	M	MIX	CR	15	30	A-NED
9	39	F	HV	CR	9	26	A-NED
10	24	M	HV	CR	38	22	A-NED
11	52	M	HV	CR	23	20	A-NED
12	51	F	HV	CR	11	3	A-NED
13	29	M	HV	CR	14	2	A-NED
14	42	F	HV	CR	16	17	A-NED

*A*-*NED* alive and no evidence of disease, *CR* complete resection, *F* female, *M* male, *HV* hyaline vascular type, *MIX* mixed type

No patients were preoperatively suspected of CD, but other diseases. No patients received any steroids or immunotherapy because no one was suspected of autoimmune diseases. All patients underwent surgical resection. Preoperative biopsy for a definitive diagnosis was excluded in concern of the deep position and the possibility of hemorrhea. Preoperation diagnosis and surgical information are summarized in (Table 2). The mean operation time was 137 min with a range of 72–472 min. The mean blood loss was 143 ml (range 50–500 ml). Three patients had a surgical approach of laparoscopy. The two of them underwent laparoscopic transperitoneal approach and the other one underwent laparoscopic retroperitoneal approach. Distal pancreatectomy was carried out unitedly in the two patients because of a preoperation misdiagnosis of occupation of pancreas. Only one patient received blood transfusion and he was sent to intensive care unit (ICU) because of massive hemorrhage. The reason of the other patient who was also sent to ICU was postoperative myasthenic crisis. The mean hospital stay was 17.9 days with a mean postoperation hospital stay of 9.2 days. No patients received chemotherapy or radiotherapy after surgical resection.

**Table 2:** Preoperation diagnosis and surgical information

Patient (*n*)	Preoperation diagnosis	Greatest diameter of lesion (cm)	Operation time (min)	Blood loss (ml)	Surgical approach	Transfer to ICU	Postoperation hospital stay (days)
1	Occupation of pancreas	6	87	100	O-TA	N	9
2	Occupation of hepatic caudate lobe	6	105	150	O-TA	N	10
3	Occupation of right adrenal	6	127	100	L-RA	N	6
4	Ectopic pheochromocytoma	3	135	100	L-RA	N	7
5	Retroperitoneal tumor	3	134	100	O-TA	N	9
6	Occupation of pancreas	4.2	72	150	O-TA	N	10
7	Retroperitoneal tumor	5.5	80	50	O-RA	N	8
8	Retroperitoneal tumor	5.5	125	100	O-TA	N	9
9	Retroperitoneal tumor	6	121	100	O-TA	N	6
10	Occupation of pancreas	7.5	472	200	L-TA	N	24
11	Occupation of right adrenal	7.5	159	500	O-TA	Y	9
12	Retroperitoneal tumor	5.5	107	100	O-TA	N	6
13	Retroperitoneal tumor	5	116	200	O-TA	Y	7
14	Retroperitoneal tumor	4	80	50	O-TA	N	9

*O*-*TA* open transperitoneal approach, *O*-*RA* open retroperitoneal approach, *L*-*TA* laparoscopic transperitoneal approach, *L*-*RA* laparoscopic retroperitoneal approach, *N* no, *Y* yes, *d* day, *ICU* intensive care unit

The mean duration of follow-up was 49.9 months (range 21–99 months). All patients received US or CT when they came back to clinic, and they no longer showed symptoms or evidence of disease after surgical resection within follow-up period. Incisional hernia happened in one patient as a complication, and hydrops in the operation area happened in two patients. No other complications were found in all the patients. All the patients had no need to take medicine of steroids or received immunotherapy after operation.

## Discussion

CD is a rare, nonneoplastic and lymphoproliferative disorder that can occur in any site where lymph nodes are present, which is a rare diagnosis in departments all over the world and remains detectable at relatively low levels. Most commonly involved sites are the mediastinum (60 %), retroperitoneum (11 %), and axilla (4 %) [3]. The etiology of CD remains unclear, although several immunological mechanisms have been proposed, including overproduction of IL-6 and human herpes virus type 8 infection [4]. Dysplastic or atypical follicular dendritic cells positive for CD21 and CD35 have frequently been described in the hyalinized center and also in the cytology smears, and can even show monoclonality, yet their role in the pathogenesis is unclear [5–7].

CD was divided into three subgroups based on its histology, which were hyaline vascular type, plasma cell type, and mixed type, and can be divided into two further forms on the basis of clinical criteria: the more common unicentric form and the less common multicentric form. The unicentric CD corresponds to the hyaline vascular variant (> 90 %). Clinically, unicentric CD tends to be present in the form of an enlarged, benign, painless lymph node that generally remains asymptomatic unless it begins to compress adjacent structures or is discovered fortuitously at the time of a routine physical examination, which occurs in young people and associated with a benign clinical course. In the current literature, unicentric CD was asymptomatic in 31 % of patients and symptomatic in 69 % of patients [8, 9], while 28.6 % patients were found as associated symptoms in our patients with unicentric retroperitoneal CD. It is difficult to differentiate CD from other tumors before pathologic diagnosis is confirmed. Some kinds of tumors which located in the retroperitoneal region commonly are listed in Table 3.

**Table 3:** Differential diagnosis

	Clinical manifestations	Pathological features	
		General features	Microscopic features
Castleman’s disease	Asymptomatic; may associated with autoimmune diseases; divided into unicentric type and multicentric type	Well-circumscribed, encapsulated mass, reddish-brown	Divided into hyaline-vascular type, plasma cell type and mixed type
Liposarcoma	Deep, painless, gradually grew tumor	Lobulated, multi-nodular, well defined	Divided into well-differentiated type, dedifferentiated type, myxoid type and pleomorphic type
Pheochromocytoma	High blood pressure, headaches, palpitations, high metabolic state, high blood sugar, sweating	Roundly, brown-yellow, internal hemorrhage, necrosis or cystic degeneration	Large irregular polygon cells, cells can be stained by chromium salts
Metastasis tumor	Different performances because of different primary tumors, lack of specificity	Various	Various

There is no definitive standard treatment regimen for unicentric CD. This study illustrated that the standard therapy of unicentric CD is surgical excision, which has been proven to be curative upon complete resection and en bloc. Complete surgical excision is the mainstay of treatment and is virtually curative in all cases reported thus far, with a 5-year survival rate approaching 100 % [2, 10]. Keller et al. reported that all their cases of unicentric CD had an indolent clinical course and mild biological behaviors, with the elimination of all systemic symptoms after complete resection [2]. Bowne et al. studied 16 cases of CD and reported that no clinical or radiographic recurrence of symptoms was found among the 8 of 16 cases of unicentric hyaline-vascular disease patients who underwent complete surgical excision [9]. There are several reports that unicentric CD in the abdominal cavity were treated laparoscopically [11, 12]. Some masses are well vascularized and adjacent to the great vessels [11]; laparoscopy can offer magnified images to facilitate and secure dissection. In our case studies, all the 14 patients with unicentric retroperitoneal CD underwent a complete surgical resection, either open or laparoscopy, and survived with excellent prognosis.

## Conclusions

In conclusion, unicentric retroperitoneal CD is a rare disease that is often misdiagnosed due to the nonspecific clinical manifestations. The final diagnosis depends on pathologic examination. Complete surgical resection of the tumor, either laparotomy or laparoscopy, is the best therapeutic alternative for unicentric CD.

### Consent

Written informed consent was obtained from the patient for publication of this case report and any accompanying images. A copy of the written consent is available for review by the Editor-in-Chief of this journal.
